# Enhanced Recovery after Surgery and Endometrial Cancers: Results from an Initial Experience Focused on Elderly Patients

**DOI:** 10.3390/cancers15123244

**Published:** 2023-06-19

**Authors:** Céline Miguet, Camille Jauffret, Christophe Zemmour, Jean-Marie Boher, Laura Sabiani, Gilles Houvenaeghel, Guillaume Blache, Clément Brun, Eric Lambaudie

**Affiliations:** 1 Department of Surgical Oncology, Institute Paoli-Calmettes, 13009 Marseille, France; celinemiguet@wanadoo.fr (C.M.);; 2 Biostatistics and Methodology Unit, Department of Clinical Research and Investigation, Institute Paoli-Calmettes, Aix Marseille University, INSERM, IRD, SESSTIM, 13009 Marseille, France; 3 Inserm, CNRS, Institute Paoli-Calmettes, CRCM, Aix Marseille University, 13009 Marseille, France; 4 Department of Anaesthesiology, Institute Paoli-Calmettes, 13009 Marseille, France

**Keywords:** enhanced recovery after surgery, endometrial cancer, length of stay, early discharge, post-operative complications, elderly, gynecological oncology surgery

## Abstract

**Simple Summary:**

The incidence of endometrial cancer is especially high among women over 70 years old. Problems can be encountered if an interest in minimally invasive surgery has been expressed, as there is a lack of evidence regarding the benefits of Enhanced Recovery After Surgery (ERAS) programs with respect to endometrial cancer management. The aim of this observational prospective study was to assess ERAS programs’ impact on length of stay, early discharge, and post-operative morbidity in the general population and among patients over 70 years old. We found a significantly shorter length of stay and a higher rate of early discharge with similar rates of post-operative complications in the group associated with the ERAS pathway. These results were even more significant among the elderly.

**Abstract:**

Endometrial cancer is the fifth most common cancer among French women and occurs most frequently in the over-70-year-old population. Recent years have seen a significant shift towards minimally invasive surgery and Enhanced Recovery After Surgery (ERAS) protocols in endometrial cancer management. However, the impact of ERAS on endometrial cancer has not been well-established. We conducted a prospective observational study in a comprehensive cancer center, comparing the outcomes between endometrial cancer patients who received care in an ERAS pathway (261) and those who did not (166) between 2006 and 2020. We performed univariate and multivariate analysis. Our primary objective was to evaluate the impact of ERAS on length of hospital stay (LOS), with the secondary objectives being the determination of the rates of early discharge, post-operative morbidity, and rehospitalization. We found that patients in the ERAS group had a significantly shorter length of stay, with an average of 3.18 days compared to 4.87 days for the non-ERAS group (estimated decrease −1.69, *p* < 0.0001). This effect was particularly pronounced among patients over 70 years old (estimated decrease −2.06, *p* < 0.0001). The patients in the ERAS group also had a higher chance of early discharge (47.5% vs. 14.5% in the non-ERAS group, *p* < 0.0001), for which there was not a significant increase in post-operative complications. Our study suggests that ERAS protocols are beneficial for the management of endometrial cancer, particularly for older patients, and could lead to the development of ambulatory pathways.

## 1. Introduction

Endometrial cancer is the fifth most common cancer among women in France, for which the mean age at diagnosis is 68 years old [1]. Its incidence rate is increasing, with obesity being one of its risk factors [2]. From 1990 to 2018, it increased mainly in the 70-year-old-and-over population (+1.4%), with maximal incidence occurring from 70 to 74 years old (93.4/100,000) [3]. The surgical management of endometrial cancer has undergone major changes following new European recommendations published by ESGO/ESTRO/ESP in 2015 [4] and updated in 2021 [5]. While the place of minimally invasive surgery (MIS) in cervical cancer treatment is still debated since the publication of the LAAC trial by Ramirez et al. [6], the place of MIS in early-stage endometrial cancer is well established [7,8], even among the elderly [9]. In addition, clinical pathways have been improved with the introduction of enhanced recovery after surgery (ERAS) [10]. To our knowledge, no studies have been conducted specifically on ERAS pathways in endometrial cancer. This study aims to evaluate whether ERAS pathways are still beneficial for patients with endometrial cancer, particularly among the elderly, or if new surgical procedures and guidelines have rendered them obsolete. 

Our primary objective was to decrease the average length of stay (LOS) for patients. Our secondary objectives were to increase the number of patients who are discharged early, defined as a target LOS of 2 days, and maintain similar rates of post-operative morbidity and rehospitalization.

## 2. Materials and Methods

### 2.1. Study Design 

This prospective observational study was conducted at the Paoli-Calmettes Institute (Marseille, France), which is a comprehensive cancer center. All consecutive patients who underwent surgery for endometrial cancer between 2006 and 2020 were identified. Inclusion criteria for participation in this study were being over the age of 18 and having been diagnosed with stage 1 or 2 endometrial cancer (according to the FIGO classification (Appendix A)) that was being surgically managed. The type of surgical procedure could be hysterectomy and/or pelvic or lombo-aortic lymphadenectomy. An omentectomy could be performed, as it is recommended for some non-endometrioid stage 1 or 2 cancers. To confirm their interest in ERAS in terms of length of hospital stay, early discharge, and post-operative complications, the first 261 patients being treated for endometrial cancer in the ERAS pathway were compared with the 166 patients being treated for the same pathology right before the implementation of the ERAS protocol in our department. The following parameters were analyzed: age, Body Mass Index (BMI), ASA (American Society of Anesthesiologists; Schaumburg, IL, USA) score, surgical procedures, surgical approaches, laparoconversion (if conducted), operative time, LOS (defined as post-operative nights spent at hospital), post-operative complications, and rehospitalization (if it occurred).

We performed subgroup analysis for patients over 70 years old. Our ERAS guidelines, which have been previously published [11], were followed by all staff involved in the treatment of patients in the ERAS pathway (including nurses, anesthesiologists, and surgeons). Data on post-operative complications and rehospitalization were collected up to one month after hospital discharge. The severity of post-operative complications was determined using the Clavien–Dindo classification [12]. Based on a review of the literature, a target LOS of 2 days was used to define an early discharge [13,14,15,16]. All procedures involving human participants in this study were conducted in accordance with French ethical standards and the 2008 Helsinki declaration. All included patients provided written informed consent before surgery. 

This work was approved by our institutional review board (IPC Comité d’Orientation Stratégique—IPC 2022-017 (RAAC-ENDO)).

### 2.2. Statistical Analysis

All statistical analyses were performed at an α = 0.05 significance level using the SAS^®^ 9.4 software. Patients’ characteristics were summarized using counts (percentages) for categorical variables and means (standard deviations) or median [min–max] for quantitative variables. Associations between groups were assessed using Chi-square or Fisher’s exact tests for small cohorts, as appropriate. For continuous variables, Wilcoxon–Mann–Whitney tests were performed. Univariate and multivariate generalized linear models were used to assess, both in the global and over-70-year-old populations, the impact of the following factors on length of stay: ERAS group, ASA 3 vs. 1–2, BMI (<25 vs. [25–30) vs. ≥30), operative time, procedure (hysterectomy and lymphadenectomy versus hysterectomy versus lymphadenectomy versus other procedures), surgery (open versus laparoscopy), and age (≥70 years old versus <70 years old, which was only applicable in the global population). Associated contrasts coefficients were estimated, using their bilateral Student’s confidence intervals and tests to determine significance.

The same parameters were also assessed through univariate and multivariate logistic regressions for predicting early discharge. Associated Odds Ratios (OR) were estimated, using their bilateral Wald’s confidence intervals and tests to determine significance.

## 3. Results

### 3.1. Patient Characteristics 

The patients’ baseline characteristics are described in Table 1. A total of 427 patients were identified and enrolled in the study, 166 of whom were enrolled in the ERAS program (from January 2006 to August 2015) and 261 of whom were not (from September 2015 to November 2020). In these two groups, 255 patients were under 70 years old and 172 were 70 years old or more. 

Age, BMI, ASA score, surgical approach, conversion to open, operative time, post-operative complications and their grade, and rehospitalization within 1 month of follow-up did not significantly differ between the two study groups.

The types of procedure significantly differed between the two populations: in the ERAS group, the patients were more commonly treated via hysterectomy only (53.2%), while the group treated prior to the implementation of the ERAS protocol more frequently underwent hysterectomy associated with lymphadenectomy (65.7%) (*p* < 0.0001).

### 3.2. Length of Stay (LOS)

The results of the univariate analysis showed that the patients in the ERAS group had a shorter LOS compared to the patients in the non-ERAS group. The average LOS was 3.18 days in the ERAS group, while it was 4.87 days in the non-ERAS group (estimated decrease −1.69 [−2.23, −1.15], *p* < 0.0001, Table 2a). The multivariate analysis confirmed ERAS was a factor that independently reduces LOS (*p* < 0.0001, Table 2a).

This reduction was even more significant among patients over 70 years old, with an average LOS of 3.37 days in the ERAS group and an average LOS of 5.43 days in the non-ERAS group (estimated decrease −2.06 [−3.03, −1.08], *p* < 0.0001, Table 2b). This association remained after multivariate analysis (*p* = 0.02, Table 2b).

Factors other than ERAS also had a significant impact on LOS. An ASA score equal to three or performing a procedure including omentectomy increased length of stay, while undergoing a minimally invasive approach decreased it (Table 2a,b).

### 3.3. Early Discharge

In the general population, being in the ERAS pathway led to a significantly higher chance of early discharge: 47.5% vs. 14.5% in the non-ERAS group (OR = 5.35 [3.26, 8.79], *p* < 0.0001, Table 3a). This result was confirmed in the multivariate analysis (OR = 5.64 [2.98, 10.68], *p* < 0.0001, Table 3a).

In the over-70-years-old group, the association between the ERAS pathway and early discharge remained significant (40.5% vs. 9.8% in the non-ERAS group, OR = 6.25 [2.48, 15.74], *p* = 0.0001, Table 3b).

This result was confirmed in the multivariate analysis (3.48 [1.20, 10.03], *p* = 0.02, Table 3b).

### 3.4. Post-Operative Complications (Table 1)

The complication rates did not differ between the two groups; 19.7% of the patients before the implementation of ERAS experienced a complication versus 15.7% in the ERAS group (*p* = 0.285).

In all, 72.6% of all complications were minor ones.

In the ERAS group, complications occurred for 41 patients, 28 of which were minor (68.3%). In the non-ERAS group, complications occurred for 32 patients, among which 25 were minor (78.1%, *p* = 0.432).

## 4. Discussion

It is already widely accepted that ERAS is feasible and safe in terms of oncological indications [11,17] and for older patients [18]. Minimally invasive surgery became the gold standard after the decrease in morbidity and increase in quality of life demonstrated by studies and meta-analysis [19,20,21]. The present study supports this position, demonstrating that in early-stage endometrial cancer, the ERAS pathway is associated with a decrease in LOS without an increase in morbidity or readmission rates, even in a high-volume cancer center with a high rate of minimally invasive surgery (e.g., where 83.1% of surgeries are performed via laparoscopy (Table 1)). LOS varies in different studies. In 2006, Marx et al. found that LOS has been reduced to 5 days when focusing on ovarian malignancy [22], while in 2014, De Groot et al. found the same result, although they studied all types of gynecologic cancers [17]. In 2008, Chase et al. found that LOS had been reduced to 2 days when focusing on endometrial carcinoma [23]. By finding that LOS had been reduced to 3 days, the results of our study are consistent with the literature. A significantly higher proportion of patients (47.5%) in the ERAS pathway were discharged on post-operative day 2 or before. In 2009, Walker et al. found that 48% of patients were discharged early in a laparoscopy group compared with a group treated via laparotomy for the comprehensive surgical staging of uterine cancer [16]. We did not find figures on early discharge within the ERAS pathway in the literature. 

Recent studies have shown that old age leads to the reception of different treatments compared to the general population [24,25].

In our study, the effect of ERAS on LOS and early discharge appeared even more significant among the elderly. Inherent attributes of the ERAS protocol such as a free diet by post-operative day 0 might explain the superior results for the over-70-years-old group, as studies have demonstrated the importance of nutrition to health status among the geriatric population [26]. Opioid-sparing analgesia might also be an explanation, as it is known that there is a higher risk of adverse effects of opioid use in geriatric pain management [27]. As it allows patients to quickly return to their everyday surroundings, the elderly may benefit the most from ERAS.

The new guidelines are in favor of surgical de-escalation (fewer lymphadenectomies with the recommendation of the sentinel node technique), and this might explain the difference in the types of procedure between the group before the implementation of the ERAS protocol, in which there were more hysterectomies with lymphadenectomy, and the group in the ERAS pathway, in which “hysterectomy only” is the most common procedure. LOS might be impacted by these differences in types of lymph node staging or surgical approaches since laparotomy and lymphadenectomy increase the risk of pre- and post-operative complications. If the distribution of surgical approaches was similar between both groups (the rate of laparoscopy is historically high in our practice), changes in our practice concerning lymph node staging were introduced in 2015, which have mainly interested the ERAS group, as the ERAS program was implemented in 2016.

However, as our main objective was to analyze the role of ERAS in LOS, we performed a multivariate analysis, which showed that ERAS is an independent factor with respect to decreases in LOS.

Despite its careful methodology, our study has some limits. 

We collected postoperative complication data up to 1 month after hospital discharge, which did not allow us to study the long-term effect of the ERAS protocol.

In our study, the complication rate is slightly higher than in some other studies [28], which is probably due to the data collection method prospectively performed at our institute by trained nurses who called the patients at days 7 and 30, and the fact that we collected data for all grades of complications. This hypothesis is supported by the fact that our rehospitalization rate is similar to that reported in the literature. 

It was also a non-randomized study, but we limited confusion bias by performing multivariate analysis on factors classically reported in the literature. Although we did not find any evidence in the literature, we can surmise that LOS might be affected by several other factors, such as postoperative first meal time, time of first mobilization, time of resumption of intestinal motility, or the drainage situation.

Preceding the adoption of ERAS protocols, many of these perioperative factors were left to individual surgeons’ discretion. The implementation of ERAS has allowed for the standardization of care, thereby providing patients with consistent perioperative management.

Our study also has strengths, such as its use of a prospective database and the expertise of our Institute, which allowed us to draw conclusions regarding the benefits of ERAS in a center where the practice of minimally invasive surgery and the ERAS pathway have already been extended.

In our study, patients in the ERAS pathway stayed 3 days on average in postoperative hospitalization, which is already short, but we wonder if the treatment we provide them cannot be provided at home by nurses.

Some studies already demonstrated the feasibility and safety of same-day discharge after hysterectomy executed alone or associated with other procedures for benign or malignant indications in well-screened patients [29,30].

The current constraints in France are due to the lack of availability of operating rooms and hospital beds. Therefore, the discovery of factors that decrease length of stay or increase the number of patients in ambulatory pathways, without increasing the number of complications and the incidence of rehospitalization, appears to be necessary.

## 5. Conclusions

Although the surgical management of endometrial cancer has changed in the last few years, our results indicate that inclusion in an ERAS protocol was beneficial for our patients and was even more important for those over 70 years old as it is an independent factor of reducing length of stay, leading to a higher proportion of early discharge without increasing the rate of post-operative complications.

These findings associated with surgical de-escalation in the management of endometrial cancer could lead to the development of ambulatory pathways. Further investigation is necessary to fully evaluate not only the feasibility but also the patient experience and satisfaction of same-day discharge after endometrial cancer surgery.

## Figures and Tables

**Table 1:** Baseline characteristics.

Parameters	Statistics	Global Population (*n* = 427)	No-ERAS (*n* = 166)	ERAS (*n* = 261)	*p*-Value *
Age	Median [Min–Max]	67 [27–91]	65.5 [40–89]	67 [27–91]	0.372
	<70 ans	255 (59.7%)	105 (63.3%)	150 (57.5%)	0.235
	≥70 ans	172 (40.3%)	61 (36.7%)	111 (42.5%)	
BMI	Median [Min–Max]	26.9 [14–63]	26.4 [14–53]	27.3 [16–63]	0.557
	<25	158 (38%)	60 (38.7%)	98 (37.6%)	0.806
	[25–30)	105 (25.2%)	41 (26.5%)	64 (24.5%)	
	≥30	153 (36.8%)	54 (34.8%)	99 (37.9%)	
ASA score	Median [Min–Max]	2 [1–3]	2 [1–3]	2 [1–3]	0.363
	1–2	342 (81%)	133 (82.6%)	209 (80.1%)	0.519
	3	80 (19%)	28 (17.4%)	52 (19.9%)	
Surgery	Laparotomy	72 (16.9%)	34 (20.5%)	38 (14.6%)	0.111
	Laparoscopy	355 (83.1%)	132 (79.5%)	223 (85.4%)	
Conversion	No	410 (96.5%)	155 (94.5%)	255 (97.7%)	0.083
	Yes	15 (3.5%)	9 (5.5%)	6 (2.3%)	
Procedure	Hysterectomy	183 (42.9%)	44 (26.5%)	139 (53.2%)	<0.0001
	Hysterectomy + lymphadenectomy	159 (37.2%)	109 (65.7%)	50 (19.2%)	
	Lymphadenectomy	42 (9.8%)	1 (0.6%)	41 (15.7%)	
	Others	43 (10.1%)	12 (7.2%)	31 (11.9%)	
Operative time	Median [Min–Max]	164 [57–551]	180 [60–480]	154 [57–551]	0.05
	Mean (SD **)	186.42 (85.44)	197.31 (88.81)	180.45 (78.3)	
Length of stay	Median [Min–Max]	3 [0–30]	4 [1–30]	3 [0–15]	<0.0001
	Mean (SD **)	3.8 (2.9)	4.9 (3.4)	3.2 (2.3)	
Early discharge	No	279 (65.3%)	142 (85.5%)	137 (52.5%)	<0.0001
	Yes	148 (34.7%)	24 (14.5%)	124 (47.5%)	
Post-operative complications	No	350 (82.7%)	130 (80.3%)	220 (84.3%)	0.285
	Yes	73 (17.3%)	32 (19.7%)	41 (15.7%)	
Grade (Clavien–Dindo)	Minor	53 (72.6%)	25 (78.1%)	28 (68.3%)	0.432
	Major	20 (27.4%)	7 (21.9%)	13 (31.7%)	
Rehospitalization	No	403 (94.6%)	159 (96.4%)	244 (93.5%)	0.201
	Yes	23 (5.4%)	6 (3.6%)	17 (6.5%)	

* Wilcoxon, Chi-square, or Fisher’s exact tests. ** Standard deviation.

**Table 2:** (**a**): Univariate and multivariate analysis of LOS in global population. (**b**): Univariate and multivariate analysis of LOS in the ≥70-year-old population.

**(a)**				
	**Univariate Analysis**		**Multivariate Analysis**	
**Parameters**	**Estimation in Days** [95% CI *]	*p*-Value **	**Estimation in Days** [95% CI *]	*p*-Value **
ERAS vs. no-ERAS	−1.69 [−2.23; −1.15]	<0.0001	−1.38 [−1.91; −0.86]	<0.0001
ASA 3 vs. 1–2	1.16 [0.46; 1.87]	0.001	1.24 [0.65; 1.83]	<0.0001
Age ≥ 70 years old vs. <70 years old	0.43 [−0.13; 0.99]	0.13	0.45 [−0.02; 0.91]	0.06
BMI [25–30) vs. <25	0.55 [−0.16; 1.27]	0.13	0.35 [−0.22; 0.91]	0.23
BMI ≥ 30 vs. <25	0.03 [−0.61; 0.68]	0.92	−0.12 [−0.65; 0.42]	0.67
Operative time	0.0004 [−0.0002; 0.001]	0.19	0.0004 [−0.0016; 0.0016]	0.89
Hysterectomy + lymphadenectomy vs. Hysterectomy only	1.38 [0.81; 1.96]	<0.0001	0.77 [0.21; 1.33]	0.008
Lymphadenectomy vs. Hysterectomy only	−0.22 [−1.12; 0.68]	0.63	0.19 [−0.58; 0.95]	0.63
Omentectomy vs. Hysterectomy only	3.39 [2.50; 4.28]	<0.0001	2.26 [1.48; 3.04]	<0.0001
Laparotomy vs. Laparoscopy	−4.22 [−4.83; −3.60]	<0.0001	−3.83 [−4.47; −3.20]	<0.0001
**(b)**				
	**Univariate Analysis**		**Multivariate Analysis**	
**Parameters**	**Estimation in Days** [95% CI *]	*p*-Value **	Estimation in Days [95% CI *]	*p*-Value **
ERAS vs. no-ERAS	−2.06 [−3.03; −1.08]	<0.0001	−1.29 [−2.32; −0.25]	0.02
ASA 3 vs. 1–2	1.22 [0.12; 2.33]	0.03	1.61 [0.59; 2.63]	0.002
BMI [25–30) vs. <25	0.23 [−1.04; 1.49]	0.73	0.53 [−0.53; 1.60]	0.32
BMI ≥30 vs. <25	−0.32 [−1.52; 0.87]	0.59	−0.51 [−1.59; 0.57]	0.35
Operative time	0.0001 [−0.0009; 0.001]	0.89	0.0002 [0.0006; 0.001]	0.68
Hysterectomy + lymphadenectomy vs. Hysterectomy only	1.07 [0.02; 2.13]	0.047	0.60 [−0.49; 1.70]	0.28
Lymphadenectomy vs. Hysterectomy only	−0.66 [−2.32; 0.99]	0.43	0.11 [−1.38; 1.61]	0.88
Omentectomy vs. Hysterectomy only	3.44 [1.93; 4.95]	<0.0001	2.78 [1.36; 4.20]	0.0002
Laparotomy vs. Laparoscopy	−4.01 [−5.21; −2.81]	<0.0001	−4.02 [−5.32; −2.73]	<0.0001

* Confidence Interval. ** Student’s *t*-test for significance.

**Table 3:** (**a**): Univariate and multivariate analyses of early discharge in the general population. (**b**): Univariate and multivariate analyses of early discharge in the ≥70-year-old population.

**(a)**				
	**Univariate Analysis**		**Multivariate Analysis**	
**Parameters**	**Odds Ratio** [95% CI *]	*p*-Value **	**Odds Ratio** [95% CI *]	*p*-Value **
ERAS vs. No-ERAS	5.35 [3.26; 8.79]	<0.0001	5.64 [2.98; 10.68]	<0.0001
ASA 3 vs. 1–2	0.48 [0.27; 0.85]	0.01	0.30 [0.15; 0.63]	0.001
Age ≥70 years old vs. <70 years old	0.69 [0.45; 1.04]	0.07	0.66 [0.39; 1.12]	0.12
BMI [25–30) vs. <25	0.47 [0.27; 0.81]	0.007	0.38 [0.20; 0.75]	0.005
BMI ≥ 30 vs. <25	0.83 [0.52; 1.30]	0.41	1.02 [0.55; 1.87]	0.96
Operative time	0.99 [0.987; 0.993]	<0.0001	0.993 [0.988; 0.998]	0.002
Hysterectomy + lymphadenectomy vs. Hysterectomy only	0.29 [0.18; 0.47]	<0.0001	0.79 [0.38; 1.62]	0.52
Lymphadenectomy vs. Hysterectomy only	1.31 [0.67; 2.56]	0.44	1.29 [0.57; 2.93]	0.54
Omentectomy vs. Hysterectomy only	0.08 [0.02; 0.27]	<0.0001	0.26 [0.06; 1.15]	0.08
Laparotomy vs. Laparoscopy	24.45 [5.90; 101.28]	<0.0001	17.33 [3.93; 76.37]	0.0002
**(b)**				
	**Univariate Analysis**		**Multivariate Analysis**	
**Parameters**	**Odds Ratio** [95% CI *]	*p*-Value **	**Odds Ratio** [95% CI *]	*p*-Value **
ERAS vs. No- ERAS	6.25 [2.48; 15.74]	0.0001	3.48 [1.20; 10.03]	0.02
ASA 3 vs. 1–2	0.50 [0.22; 1.13]	0.10	0.30 [0.11; 0.81]	0.02
Age ≥ 70 years vs. <70 years	0.69 [0.29; 1.63]	0.40	0.51 [0.19; 1.36]	0.18
BMI [25–30) vs. <25	1.13 [0.53; 2.42]	0.75	1.44 [0.55; 3.74]	0.46
BMI ≥ 30 vs. <25	0.993 [0.989; 0.998]	0.009	0.997 [0.990; 1.004]	0.35
Operative time	0.29 [0.12; 0.67]	0.004	0.65 [0.21; 2.00]	0.45
Hysterectomy + lymphadenectomy vs. Hysterectomy only	1.93 [0.65; 5.70]	0.24	1.40 [0.41; 4.74]	0.59
Lymphadenectomy vs. Hysterectomy only	0.08 [0.01; 0.62]	0.02	0.12 [0.01; 1.23]	0.07
Omentectomy vs. Hysterectomy only	13.68 [1.80; 103.82]	0.01	12.22 [1.45; 102.79]	0.02

* Confidence Interval. ** Wald test for significance.

## Data Availability

All data can be found in the text.

## Appendix A. FIGO Classification

**Stage**	**Tumoral Invasion**
Stage I	Tumor confined to uterus
IA	<50% myometrial invasion
IB	>50% myometrial invasion
Stage II	Cervical stromal invasion
Stage III	Local and regional spread
IIIA	Invasion into adnexa or serosa
IIIB	Vaginal or parametrial involvement
IIIC	Node involvement
IIIC1	Pelvic node involvement
IIIC2	Para-aortic lymph node involvement
Stage IV	Distal invasion
IVA	Tumoral invasion of bladder and/or bowel mucosa
IVB	Distant metastases including inguinal nodes and abdominal metastases
